# Comorbidities in Chronic Spontaneous Urticaria: Integrating Disease Drivers and Systemic Consequences

**DOI:** 10.3390/ijms27219615

**Published:** 2026-10-27

**Authors:** Joo-Hee Kim, Mi-Ae Kim, Young-Min Ye

**Affiliations:** 1 Division of Pulmonary, Allergy, and Critical Care Medicine, Department of Internal Medicine, Hallym University Sacred Heart Hospital, Hallym University College of Medicine, Anyang 14068, Republic of Korea; luxjhee@hallym.or.kr; 2 Department of Pulmonology, Allergy and Critical Care Medicine, CHA Bundang Medical Center, CHA University, Seongnam 13496, Republic of Korea; makim@cha.ac.kr; 3 Department of Allergy and Clinical Immunology, Ajou University School of Medicine, 164 WorldCup-ro, Yeongtong-gu, Suwon 16499, Republic of Korea

**Keywords:** chronic spontaneous urticaria, comorbidities, mast cell activation, autoimmunity, atopy, psychiatric disorders, neuroimmune interactions, anaphylaxis

## Abstract

Chronic spontaneous urticaria (CSU) is a mast cell-driven inflammatory skin disorder traditionally considered self-limiting and non-life-threatening. However, accumulating evidence suggests that CSU is associated with multiple comorbidities, reflecting systemic immune and neuroinflammatory dysregulation. These comorbidities may act as both triggers and consequences of disease activity, forming a complex network centered on mast cell activation. Autoimmune diseases, particularly autoimmune thyroid disease, are strongly associated with CSU, especially in the type IIb autoimmune endotype. By contrast, type I autoallergic CSU is associated with immunoglobulin E (IgE) autoantibodies. Atopic diseases, neuroimmune interactions, and psychiatric conditions may further contribute to disease persistence through mechanisms involving IgE signaling and substance P-mediated mast cell activation via Mas-related G protein-coupled receptor X2. Metabolic and cardiovascular comorbidities have also been reported, with recent large-scale cohort studies strengthening the evidence for an association with cardiovascular risk, although causality remains unestablished. Associations with malignancy remain uncertain, whereas recent real-world studies suggest increased mortality among patients with CSU, potentially reflecting the underlying comorbidity burden rather than direct disease effects. Importantly, effective disease control may improve CSU outcomes and associated comorbidities. A comprehensive, mechanism-based approach that integrates comorbidity assessment into clinical management is essential for optimizing patient care.

## 1. Introduction

Chronic spontaneous urticaria (CSU) is a common, predominantly mast cell-driven inflammatory skin disorder characterized by recurrent wheals, angioedema, or both, persisting for >6 weeks [1]. Although its clinical manifestations are predominantly cutaneous, CSU is increasingly recognized as a heterogeneous inflammatory disorder associated with a broad spectrum of comorbidities, including autoimmune, atopic, psychiatric, cardiometabolic, infectious, and other systemic conditions (Table 1) [1,2,3].

The clinical relevance of these comorbidities extends beyond their coexistence with CSU. Some may reflect shared genetic or immunologic susceptibility, others may modify mast cell activation or disease activity, and still others may develop as consequences of persistent inflammation, treatment exposure, or the substantial psychosocial burden of chronic disease [2,38]. Importantly, these relationships are unlikely to be uniform across patients. CSU itself comprises distinct but overlapping pathogenic pathways, including immunoglobulin E (IgE)-mediated autoallergy, type IIb autoimmunity, and non-IgE-mediated mast cell activation [39]. Comorbidity patterns may therefore provide clinically accessible clues to the underlying inflammatory milieu and disease endotype.

Autoimmune disease provides the clearest example of such a mechanistic association. Autoimmune thyroid disease and several other organ-specific and systemic autoimmune disorders occur more frequently in patients with CSU, particularly those with features of type IIb autoimmune CSU [3]. Atopic diseases may characterize a different clinical and immunologic background, although atopy or elevated total IgE alone should not be considered evidence of type I autoallergic CSU. Psychiatric disorders illustrate another dimension of these relationships, with chronic itch and sleep disturbance interacting with stress-responsive and neuroimmune pathways [2]. Cardiometabolic diseases may further reflect or amplify systemic low-grade inflammation, whereas reported associations with infections and malignancies raise different questions about causality, disease modification, and clinical surveillance [6].

**Figure 1:**
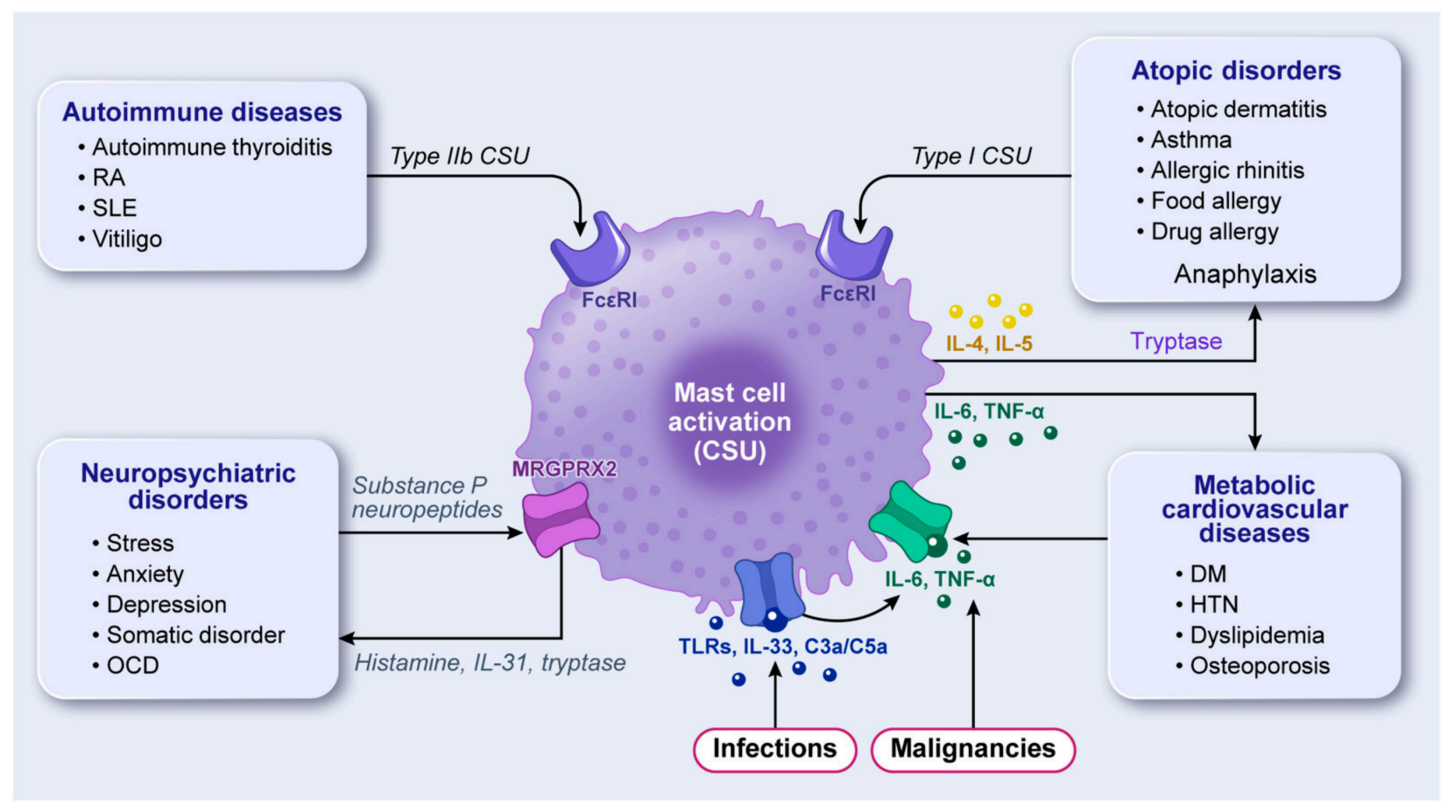
Proposed mast cell-centered framework linking comorbidities with chronic spontaneous urticaria. This schematic illustrates a proposed mast cell-centered framework through which autoimmune, atopic, neuroimmune, infectious, and metabolic/vascular factors may interact with CSU and its associated comorbidities. CSU is a mast cell-driven condition involving multiple activating and amplifying pathways. In type IIb autoimmune CSU, functional IgG autoantibodies against FcεRIα or IgE can activate mast cells and basophils, whereas autoallergic and atopic pathways involve autoreactive and allergen-specific IgE, respectively, acting through FcεRI signaling. Neuroimmune signals, including substance P, may modulate mast cell activation through MRGPRX2 and other pathways, providing a potential interface among psychological stress, sensory nerves, and CSU. Infections may influence mast cell activation through innate immune and alarmin pathways, including Toll-like receptors (TLRs), IL-33/ST2, and complement signaling. Metabolic disorders, including obesity and MetS, are associated with chronic low-grade inflammation, endothelial dysfunction, and coagulation abnormalities, which may interact with inflammatory and vascular pathways implicated in CSU. Activated mast cells release histamine, tryptase, cytokines, and other mediators that can affect vascular, immune, and neural responses, providing potential amplification loops between CSU and its comorbid conditions. Collectively, these interconnected processes may link underlying disease drivers and modifiers with broader systemic manifestations and comorbidity burden, including susceptibility to anaphylaxis and neuropsychiatric comorbidities. Bidirectional interactions between mast cell activation and comorbid conditions may contribute to disease persistence, treatment refractoriness, and the clinical heterogeneity of CSU. However, while several individual pathways of mast cell activation are supported by experimental evidence, their proposed connections with CSU-associated comorbidities remain largely hypothetical and require further investigation. CSU, chronic spontaneous urticaria; DM, diabetes mellitus; HTN, hypertension; IgE, immunoglobulin E; IgG, immunoglobulin G; IL, interleukin; MetS, metabolic syndrome; MRGPRX2, Mas-related G protein-coupled receptor X2; OCD, obsessive–compulsive disorder; RA, rheumatoid arthritis; SLE, systemic lupus erythematosus; ST2, suppression of tumorigenicity 2; TNF, tumor necrosis factor.

Therefore, comorbidities in CSU should not be viewed simply as a list of coincidental diagnoses. Their type, timing, and relationships with disease activity and treatment response may help reveal the biological heterogeneity of CSU while also defining its broader systemic burden. Previous reviews have summarized the epidemiological associations between CSU and various comorbidities and discussed their potential clinical implications [6]. Building on this literature, the present review places greater emphasis on distinguishing shared disease susceptibility, potential modifiers of CSU activity, and downstream consequences, while examining the strength and limitations of the supporting evidence. By integrating epidemiological findings with current concepts of mast cell activation and CSU endotypes, we propose a conceptual framework for understanding the potential relationships between CSU and its associated comorbidities (Figure 1) and discuss the implications for individualized clinical assessment and management.

## 2. Autoimmune Comorbidities and Autoimmune Endotypes

Autoimmunity represents one of the most consistently documented systemic comorbidities associated with CSU. Patients with CSU have increased rates of organ-specific and systemic autoimmune diseases, including autoimmune thyroid disease, vitiligo, rheumatoid arthritis, systemic lupus erythematosus, type 1 diabetes, and pernicious anemia [3,5].

Among these, autoimmune thyroid disease is the most extensively studied and provides an important link between clinical comorbidity and CSU endotype [3,5,33]. Anti-thyroid peroxidase (TPO) antibodies are the most consistently reported thyroid autoantibodies in CSU [3]. A meta-analysis of case–control studies including >14,000 patients with chronic urticaria (CU) demonstrated approximately fivefold higher odds of anti-TPO positivity than in controls [4]. Hashimoto thyroiditis and hypothyroidism are more commonly associated with CSU than Graves’ disease and hyperthyroidism [3]. Importantly, thyroid autoimmunity may be present even in euthyroid patients, indicating that the association cannot be explained solely by altered thyroid hormone levels [40]. The relationship between thyroid autoimmunity and CSU is particularly relevant to type IIb autoimmune CSU [41]. In this endotype, mast cell and basophil activation is mediated by functional autoantibodies, most commonly immunoglobulin G (IgG) directed against the α-chain of the high-affinity IgE receptor (FcεRIα) or IgE. When defined using strict criteria, the combination of a positive autologous serum skin test (ASST), demonstrable IgG autoantibodies against FcεRIα or IgE, and a positive basophil activation or histamine-release assay identifies a relatively small but clinically distinct subgroup of patients with type IIb autoimmune CSU [41]. These patients typically have lower total IgE levels, higher IgG anti-TPO levels, basopenia, eosinopenia, greater disease severity, and a higher likelihood of concomitant autoimmune disease [3,41]. They also tend to respond more slowly or less completely to omalizumab and may respond more favorably to cyclosporine [39]. The association between thyroid autoimmunity and type IIb CSU does not necessarily imply that anti-thyroid antibodies directly induce wheals. Rather, IgG anti-TPO may serve as a marker of a broader autoimmune milieu associated with functional mast cell-activating autoantibodies [42,43]. Accordingly, anti-TPO IgG positivity alone is insufficient to define the type IIb autoimmune endotype of CSU, and thyroid autoantibody testing should be interpreted in the appropriate clinical context [44].

Importantly, autoimmunity in CSU may extend beyond autoantibodies directly targeting the IgE-FcεRIα axis. IgG autoantibodies against heat shock protein 10 have been detected more frequently in patients with CSU than in healthy controls and have been associated with greater disease activity [45]. Rather than directly cross-linking FcεRIα, this pathway may contribute to CSU through the loss of the inhibitory effects of heat shock protein 10 on platelet-activating factor-induced mast cell activation, suggesting that autoantibodies may influence disease through mechanisms distinct from classical type IIb autoimmunity [45]. Complement activation may provide an additional IgE-independent pathway of mast cell activation and may also amplify FcεRIα-mediated responses [46]. In CSU, tissue factor expressed by eosinophils and other cells may activate the extrinsic coagulation pathway, leading to thrombin generation and increased vascular permeability [46,47]. Activated coagulation factors may also promote C5 cleavage and C5a generation, which can activate skin mast cells through the C5a receptor (C5aR), thereby linking coagulation and complement activation to wheal formation [46]. In addition, C4d deposition in dermal vessels and increased circulating C5a levels have been demonstrated in CSU, suggesting that complement activation, potentially involving immune complexes, may occur in a subset of patients [48]. C5a may further amplify functional IgG-mediated mast cell and basophil activation, providing an additional mechanism relevant to type IIb autoimmune CSU [42,46,49]. Thus, autoreactivity in CSU may encompass several mechanistically distinct processes, ranging from direct FcεRIα activation to modulation of endogenous regulatory proteins and immune complex–complement pathways.

A distinct but potentially overlapping mechanism is type I autoimmune, or autoallergic, CSU, in which IgE autoantibodies recognize self-antigens and activate FcεRI-expressing mast cells and basophils [50]. IgE autoantibodies against TPO and interleukin (IL)-24 are among the best-characterized candidates, although autoreactive IgE against several other self-antigens, including thyroglobulin and tissue factor, has also been reported [51,52,53]. Type I and type IIb autoimmune mechanisms are not mutually exclusive, and substantial overlap between these two endotypes has been reported, although the true frequency and clinical significance of this overlap remain uncertain [50,54].

Importantly, however, immune responses against the same autoantigen do not necessarily generate parallel IgG and IgE responses. In studies of TPO autoimmunity, IgG anti-TPO responses were more strongly associated with autoimmune thyroid disease, whereas functionally active IgE anti-TPO was enriched in CSU [52,55]. Moreover, among patients with CSU, IgE and IgG anti-TPO frequently occurred independently and were associated with distinct clinical profiles [52]. Together, these observations suggest that autoantibody isotype and the resulting clinical phenotype may be shaped not only by the identity or abundance of the autoantigen but also by the tissue and immunologic context in which the autoantigen is encountered.

Taken together, autoimmune comorbidities in CSU may represent more than coincidental accompanying diseases. Their association with type IIb autoimmune CSU suggests that they may serve as clinical manifestations of an underlying autoimmune-prone immunologic milieu. Notably, in a large population-based study, several autoimmune diseases, including rheumatoid arthritis, Sjögren syndrome, celiac disease, type 1 diabetes, and systemic lupus erythematosus, were diagnosed predominantly within 10 years after the diagnosis of CU, suggesting that urticaria may precede the clinical recognition of broader systemic autoimmunity [5]. Although this temporal relationship does not establish causality, it supports the possibility that shared autoimmune mechanisms may manifest initially as CSU and subsequently as other organ-specific or systemic autoimmune diseases. Thus, autoimmune comorbidities may provide clinically accessible clues regarding disease-driving immune mechanisms while also representing part of the evolving systemic disease burden associated with CSU.

## 3. Atopic Comorbidities

Atopic diseases are among the most frequently reported comorbidities of CU and include allergic rhinitis (AR), asthma, atopic dermatitis (AD), and food allergy (FA) [6]. However, their prevalence and clinical patterns vary considerably across age groups and populations. Across European and Asian studies, AR is generally the most frequently reported atopic comorbidity, whereas asthma and AD are particularly prominent in children [6,7,8,9,10,11]. Reported prevalence estimates vary widely across cohorts, ranging from approximately 15% to >50% for AR, about 10–40% for asthma, and 5% to >20% for AD, depending on age, population, and the method of ascertainment [6,9,10,11,12,13,14,15]. Age-stratified studies from Korea, Japan, and China consistently indicate that patterns of atopic comorbidity vary across the lifespan: asthma and AD are relatively more prominent in younger patients, whereas AR remains common across a broader range of age groups [9,10,11]. In a diverse United States adult cohort, CSU was strongly associated with AD, AR, and asthma, even after adjustment for major autoimmune comorbidities [12]. Similarly, a cohort from Northern Israel identified concomitant AD in approximately 10% of patients with CSU, and patients with both CSU and AD more frequently had asthma and elevated total IgE levels than those with CSU alone [13]. Together, these observations suggest that atopic comorbidity in CSU reflects an age- and population-dependent type 2 inflammatory background rather than a fixed feature of a single CSU endotype.

Longitudinal observations further suggest that the relationship between atopic diseases and CSU extends beyond simple coexistence. In a pediatric cohort, a history of early AD was associated with an approximately threefold higher likelihood of subsequent CSU [56]. Conversely, longitudinal population-based studies indicate that CSU may precede the development of atopic diseases [16,17]. A Danish nationwide registry study demonstrated an increased subsequent risk of AD after CU [16]. More recently, a Korean nationwide cohort of >1.1 million individuals revealed a complex, age-dependent network of transitions among allergic diseases, with CU, AD, and asthma each acting as major precursor conditions for the subsequent development of other allergic disorders [17]. AD was associated with the later development of CU, whereas CU itself was a central precursor linking AD, anaphylaxis, and FA. These inter-disease connections were generally stronger in adults than in children and remained prominent in older adults [17]. These findings argue against a simple unidirectional “atopic march” toward CSU and instead support an age-dependent, multidirectional network of allergic susceptibility in which the clinically apparent disease may vary over the life course.

The biological basis for these associations remains incompletely understood. CSU is primarily a mast cell-driven disease rather than a conventional allergen-driven disorder, but atopic diseases and CSU share several immunologic components, including IgE, mast cells, and type 2 cytokine signaling [1,39]. IL-4 and IL-13 promote IgE class switching in B cells, whereas IL-4 can enhance FcεRI expression and IgE-mediated responsiveness in human mast cells, providing a potential interface between an atopic inflammatory environment and CSU [57,58]. Nevertheless, the presence of atopy, allergic sensitization, or elevated total IgE does not establish type I autoimmune, or autoallergic, CSU. Environmental allergen-specific IgE may simply reflect the patient’s atopic background, whereas type I autoallergic CSU requires IgE directed against self-antigens with potential functional relevance. Thus, atopy should not be regarded as a surrogate marker of type I autoallergic CSU.

Atopy may nevertheless modify the clinical expression of CSU. In a Chinese cross-sectional study, patients with atopic CSU more frequently had dermographism, angioedema, recurrent urticaria, and anaphylaxis, whereas atopy was not associated with ASST positivity, overall disease activity, disease duration, or response to antihistamines [59]. These findings raise the possibility that an atopic background influences mast cell reactivity or the threshold for systemic mediator-related manifestations rather than simply increasing CSU severity.

Anaphylaxis may represent an important clinical intersection between atopic susceptibility and mast cell hyperresponsiveness in CSU. Population-based studies have reported an increased risk of anaphylaxis among patients with urticaria compared with the general population [16,17,18]. Notably, the Korean nationwide longitudinal analysis identified CU as a major preceding condition associated with the subsequent development of anaphylaxis and FA across age groups [17]. This temporal association does not establish that CU directly causes anaphylaxis but may instead indicate a shared susceptibility to systemic mast cell activation. In this context, concomitant FA and exposure to cofactors such as nonsteroidal anti-inflammatory drugs may further lower the threshold for systemic reactions in susceptible patients. Thus, in some patients, CSU may serve as a clinical marker of an underlying propensity for broader mast cell-mediated allergic responses, although whether CSU itself contributes to the subsequent risk of anaphylaxis remains uncertain.

Taken together, atopic comorbidities in CSU may be better viewed as manifestations of a shared and evolving allergic inflammatory context rather than as evidence of a single CSU endotype. Their age-dependent and multidirectional temporal relationships suggest that pre-existing atopic disease may modify the immunologic environment in which CSU develops, whereas atopic diseases emerging after CSU may represent later manifestations of shared inflammatory susceptibility. The association of CSU with FA and anaphylaxis further suggests that in some patients, this shared background may extend beyond type 2 inflammation to an increased propensity for systemic mast cell activation. Therefore, the temporal order and clinical expression of CSU and its allergic comorbidities may reflect which component of an interconnected allergic and mast cell-mediated network becomes clinically apparent first rather than a simple unidirectional cause-and-effect relationship.

## 4. Neuroimmune Interactions and Psychiatric Comorbidities

Psychiatric comorbidities, particularly depression and anxiety, are more prevalent among patients with CU than in the general population [16,19]. A systematic review and meta-analysis estimated that approximately one in three patients with CU has at least one psychiatric disorder; however, the temporal relationship between psychiatric illness and CU onset could not be determined, and no association was observed between psychological functioning and CU severity or disease duration [19]. In Danish nationwide registry data, depression was the most frequent psychiatric comorbidity, and patients with CU had higher odds of depression than matched controls (odds ratio [OR], 1.5; 95% confidence interval [CI], 1.3–1.7) [16]. A cross-sectional study using Korean national health insurance claims data found that compared with patients with non-atopic eczema, those with CSU had significantly higher odds of anxiety (OR, 1.10; 95% CI, 1.04–1.16), sleep disorder (OR, 1.18; 95% CI, 1.11–1.26), and suicidal ideation (OR, 1.33; 95% CI, 1.15–1.53) [21]. In a Korean multicenter study using the Hospital Anxiety and Depression Scale (HADS), the prevalence of depression and anxiety among patients with CU was 48.1% and 38.0%, respectively; anxiety, depression, and perceived stress were each inversely correlated with disease-specific quality of life [20].

Beyond autoimmune and atopic mechanisms, neuroimmune interactions may contribute to CSU pathogenesis, although their precise role in disease initiation and persistence remains incompletely understood. Psychological stress may influence CSU through pathways involving cutaneous sensory nerves and neuropeptides [20,60]. Substance P can activate skin mast cells through Mas-related G protein-coupled receptor X2 (MRGPRX2), providing an IgE-independent pathway for mast cell degranulation [61]. Serum substance P levels are elevated in patients with CSU and are reportedly higher in those with comorbid depression [62,63]. Altered circulating levels of calcitonin gene-related peptide, brain-derived neurotrophic factor, and IL-31 have also been described, implicating neurogenic inflammation, neurotrophin signaling, and pruritogenic cytokine pathways [64,65,66]. Conversely, mast cell-derived histamine and tryptase can sensitize and activate sensory nerve endings, forming a bidirectional signaling network between cutaneous mast cells and sensory neurons [2,39]. At the central level, functional neuroimaging has demonstrated altered brain connectivity in patients with CSU, suggesting changes in sensory and affective processing that may link chronic itch to anxiety [67]. Although the causal significance of these findings remains uncertain, peripheral neuroimmune signaling and central processing may together sustain a self-reinforcing cycle of itch, psychological distress, and inflammation.

The relationship between CSU and psychological well-being is also reflected in treatment outcomes. Omalizumab treatment has been associated with improvements in quality of life, perceived stress, anxiety, and depressive symptoms in patients with CSU [68]. However, a case–control study found greater psychological distress and poorer sleep quality than in healthy controls even among patients with a good treatment response, suggesting that symptom control may not fully resolve the psychological burden [69]. Some antidepressants with antihistaminic properties have also shown efficacy against urticaria. A systematic review reported that doxepin, a tricyclic antidepressant with potent H_1_-receptor-blocking activity, improved chronic idiopathic urticaria in small double-blind crossover trials, with efficacy similar to that of mequitazine and greater than that of diphenhydramine [70].

Taken together, psychiatric and sleep comorbidities represent an important but incompletely characterized component of the broader burden of CU. Their relationship with CSU is likely bidirectional: psychological stress and sleep disturbance may aggravate disease through neuroimmune pathways, whereas persistent pruritus and poor disease control may, in turn, contribute to psychological distress and impaired sleep. However, systematic epidemiologic studies examining the temporal relationship between CSU and psychiatric disorders, particularly in Asian populations, remain limited. Further studies are needed to clarify their temporal sequence and shared risk factors, elucidate the underlying neuroimmune mechanisms, and determine whether improved CSU control can modify long-term psychiatric and sleep outcomes.

## 5. Metabolic and Cardiovascular Comorbidities

Metabolic abnormalities are increasingly recognized in patients with CU, although their causal relationship with urticaria remains uncertain. Obesity, metabolic syndrome (MetS), hypertension, dyslipidemia, and diabetes have been reported more frequently among patients with CU across diverse populations [7,9,22,34]. A recent systematic review and meta-analysis of 10 studies from six countries estimated a pooled MetS prevalence of 25.2% among patients with CU and approximately 1.6-fold higher odds of MetS than in controls, although the certainty of the evidence was rated as low because of serious risk of bias and inconsistency across studies [23].

Metabolic comorbidity may also be associated with CSU severity. In a Korean study, MetS was present in 29.8% of patients with CU compared with 17.8% of matched controls and was independently associated with uncontrolled urticaria [22]. Patients with both CU and MetS had higher levels of tumor necrosis factor-α, eosinophil cationic protein, and complement components, suggesting a shared proinflammatory milieu [22]. Other studies have reported associations between dyslipidemia and circulating levels of IL-6, tumor necrosis factor-α, and C-reactive protein, further supporting an overlap between metabolic inflammation and CSU-associated systemic inflammation [71,72].

Recent large-scale epidemiologic studies have further strengthened the evidence for an association between CU and cardiovascular disease. In a national cohort study using the MarketScan databases, CU was independently associated with atherosclerotic cardiovascular disease after adjustment for relevant cardiovascular risk factors [25]. This finding suggests that the observed cardiovascular burden may not be explained solely by the clustering of conventional metabolic risk factors. Nevertheless, because the available evidence is observational, a causal relationship cannot be established, and it remains unclear whether CU itself contributes directly to atherosclerotic cardiovascular disease.

Several mechanisms have been proposed to explain the association between metabolic dysfunction and CSU. Obesity and MetS are characterized by chronic low-grade inflammation, altered adipokine signaling, oxidative stress, endothelial dysfunction, and activation of coagulation pathways, several of which overlap with pathways implicated in CSU [73,74]. These abnormalities could lower the threshold for mast cell activation or amplify inflammatory and vascular responses. Conversely, persistent inflammation associated with active CSU may contribute to systemic inflammation and endothelial stress [74]. Thus, metabolic comorbidity may represent not only a pre-existing inflammatory context that modifies CSU development but also a component of a bidirectional inflammatory amplification process. However, although these shared biological features provide plausible mechanistic links, direct evidence that metabolic abnormalities promote mast cell activation or sustain CSU remains limited.

The relationship between CSU and cardiovascular disease has historically been less consistent. Earlier population-based studies did not uniformly demonstrate an increase in cardiovascular events despite the higher prevalence of conventional cardiovascular risk factors among patients with CU [22,75,76]. More recent longitudinal evidence, however, suggests that cardiovascular risk may extend beyond risk-factor clustering. In a large real-world cohort of 105,593 propensity score-matched patients with CSU and an equal number of controls, CSU was associated with an increased subsequent risk of major adverse cardiovascular events (adjusted hazard ratio [aHR], 1.25), venous thromboembolism (aHR, 1.63), conduction disorders (aHR, 1.82), and several other cardiovascular outcomes over a median follow-up of approximately 3 years [24]. Although these findings support an epidemiological association between CSU and cardiovascular outcomes, residual confounding, differences in healthcare utilization, and potential surveillance bias remain important limitations. Moreover, the observational design does not establish whether CSU-related inflammatory or coagulation abnormalities directly contribute to cardiovascular events [24].

Metabolic inflammation and vascular dysfunction may provide a host environment that influences mast cell activation and CSU disease expression. Conversely, inflammatory and vascular abnormalities observed in CSU may overlap with pathways implicated in cardiovascular disease. Whether effective CSU control can modify long-term metabolic or cardiovascular outcomes remains unknown and warrants prospective investigation.

## 6. Infections and Vaccination as Disease Triggers and Modifiers

Infections have long been investigated as potential triggers or modifiers of CSU, although their relevance appears to vary according to the pathogen and host susceptibility. Among chronic infections, *Helicobacter pylori* has been the most extensively studied. A meta-analysis of 22 studies including 1385 patients with CSU found that *H. pylori*-positive patients were less likely to show spontaneous remission and that eradication therapy was associated with a higher likelihood of CSU remission than no treatment [26]. Notably, however, improvement in CSU was not associated with successful bacterial eradication, leaving the causal contribution of *H. pylori* uncertain [26]. In contrast, an earlier systematic review reported that successful *H. pylori* eradication was associated with a higher likelihood of urticaria remission than unsuccessful eradication [27]. These conflicting findings indicate that the relationship between *H. pylori* eradication and CSU improvement remains uncertain.

By contrast, chronic viral hepatitis does not appear to be consistently associated with CSU. A systematic review of 32 studies including 5014 patients with CSU found chronic hepatitis B virus (HBV) and hepatitis C virus (HCV) in ≤5% and ≤2% of patients, respectively, in most studies [28]. Although these rates were generally comparable to those in the general population, some regional studies reported higher HCV seroprevalence, indicating geographic heterogeneity and precluding a definitive conclusion regarding the absence of an association [28]. Routine screening for HBV or HCV is therefore not supported in patients with CSU without relevant risk factors, abnormal liver function, or features suggesting urticarial vasculitis [1,28]. These contrasting observations suggest that the presence of chronic infection alone is unlikely to be sufficient to drive CSU and that pathogen-specific immune responses and host susceptibility may be more relevant than infection per se.

The COVID-19 pandemic provided further evidence that acute immune perturbations may modify the course of urticaria. SARS-CoV-2 infection has been associated with exacerbation of pre-existing CU. In an international cohort, 37% of patients with CU who developed COVID-19 experienced worsening of urticaria, with exacerbations occurring more frequently among patients requiring hospitalization [29]. Population-based evidence has also demonstrated an increased risk of new-onset CSU following COVID-19 [30,31]. A binational population-based cohort analysis from South Korea and Japan demonstrated an increased risk of incident CU after SARS-CoV-2 infection (aHR, 1.80; 95% CI, 1.64–1.98) [30]. In a subsequent Korean nationwide study including nearly 5 million patients with COVID-19 and an equal number of matched uninfected controls, SARS-CoV-2 infection was associated with a 13% higher risk of incident CU after multivariable adjustment (hazard ratio [HR], 1.13; 95% CI, 1.10–1.16) [31].

Reports of CU onset or exacerbation following vaccination suggest a possible relationship between acute immune stimulation and urticaria, although temporal associations alone do not establish causality. In a global pharmacovigilance analysis using VigiBase, 1898 reports of vaccine-associated CU were identified between 2010 and 2023, with 96.6% reported during 2020–2023 and most involving COVID-19 mRNA vaccines [32]. Disproportionality analysis showed the strongest reporting association for COVID-19 mRNA vaccines (reporting OR, 26.52; 95% CI, 24.33–28.90), although associations were also observed for papillomavirus, influenza, adenovirus-vectored COVID-19, and zoster vaccines [32]. However, these pharmacovigilance signals are subject to reporting bias, do not establish causality or provide reliable estimates of disease risk, and cannot reliably distinguish new-onset CU from relapse or exacerbation of pre-existing disease. Post-vaccination CU should not necessarily be interpreted as immediate IgE-mediated vaccine hypersensitivity. Immune profiling of patients with COVID-19 vaccine-associated CU has demonstrated increased levels of mast cell- and T cell-related cytokines, autoreactive IgE and IgG responses against IL-24 and thyroid-related antigens, and anti-polyethylene glycol IgG [77]. In another study, patients with CU relapse following COVID-19 vaccination more frequently had ASST positivity, antinuclear antibodies, and basopenia [78]. These observations suggest that vaccination may amplify pre-existing autoimmune or autoallergic pathways in susceptible individuals rather than acting solely through conventional allergen-specific IgE responses [79].

## 7. Malignancy and Mortality

The association between CSU and malignancy remains controversial [6,34,37]. Population-based studies have reported increased frequencies of selected malignancies among patients with CSU, but the magnitude and consistency of these associations vary across populations [6,33,34,37]. In a Korean nationwide study, malignancies were among the comorbidities identified in patients with CSU, with stomach, thyroid, liver, and prostate cancers commonly represented [33]. However, these epidemiologic associations do not establish causality. Differences in age, underlying comorbidity burden, healthcare utilization, and surveillance intensity may have contributed to the observed associations.

Several mechanisms have been proposed to explain the possible association between CSU and malignancy. Chronic inflammation and immune dysregulation may provide a shared biological background, whereas malignancy-associated immune responses or tumor-derived mediators could, in rare cases, promote mast cell activation and urticarial manifestations [80,81,82]. Conversely, increased healthcare contact and diagnostic activity following a diagnosis of urticaria may increase the likelihood of detecting otherwise occult malignancies [35]. Nevertheless, evidence supporting a direct mechanistic or causal relationship remains limited, and malignancy should not be considered an established driver of CSU [35,36]. Accordingly, routine malignancy screening is not warranted in patients with otherwise typical CSU without clinical features suggesting an underlying neoplastic disorder [1].

Recent evidence has also raised concerns regarding long-term health outcomes in CSU. In a large real-world propensity score-matched cohort, adults with CSU had higher all-cause mortality at 3 months, 1 year, and 5 years, along with increased risks of several clinically important conditions, including malignant neoplasms and suicidal ideation or suicide attempts [37]. These findings suggest that the health burden associated with CSU may extend beyond cutaneous symptoms, although they do not establish that CSU itself directly causes excess mortality. Rather, the observed mortality signal may reflect systemic and psychosocial comorbidity burden, residual confounding, differences in healthcare utilization, or other unmeasured factors. Notably, lower 5-year mortality was observed among patients receiving guideline-recommended CSU treatments in the same study [37]. However, differences in baseline health status, treatment selection, healthcare access, and other unmeasured factors may account for this association. These findings should therefore not be interpreted as evidence that CSU treatment reduces mortality. Whether improved CSU control can modify long-term systemic outcomes remains an important question for prospective investigation.

Taken together, current evidence does not support malignancy as a routine underlying cause of CSU, but emerging associations with malignancy and mortality highlight that CSU may coexist with clinically important systemic and psychiatric comorbidities that contribute to long-term health burden. Clarifying whether these associations reflect shared disease mechanisms, consequences of persistent inflammation, comorbidity clustering, or healthcare-related factors will require longitudinal studies specifically designed to address temporality and causality.

## 8. Treatment-Related Morbidity and Disease Burden

Not all comorbidities observed in patients with CSU necessarily reflect shared disease mechanisms. Some may arise as consequences of persistently uncontrolled disease or its treatment. Systemic corticosteroids are particularly relevant because repeated or prolonged exposure may contribute to metabolic complications, diabetes, hypertension, osteoporosis, infection, and other treatment-related morbidities [83,84]. Current treatment strategies therefore restrict systemic corticosteroids to short courses for acute exacerbations and emphasize steroid-sparing, guideline-directed therapies for long-term disease control [1].

Persistent CSU itself imposes a substantial health burden [85]. Recurrent pruritus, wheals, and angioedema can disrupt sleep, daily activities, work productivity, and social functioning, and are associated with impaired quality of life and increased healthcare utilization [86,87]. Poor disease control is also associated with a greater socioeconomic burden, including substantial productivity loss and indirect costs [88]. Importantly, disease burden may interact with psychiatric, metabolic, and other comorbidities, making it difficult to distinguish those that contribute to CSU persistence or severity from those that emerge or worsen as consequences of uncontrolled disease. Effective treatment improves quality of life and may reduce healthcare utilization and the need for potentially harmful rescue therapies. Whether sustained disease control can modify the development or course of systemic comorbidities remains unknown.

Thus, treatment exposure and uncontrolled disease should be considered when interpreting the comorbidity burden of CSU. Distinguishing disease drivers from downstream consequences may help clarify these relationships and guide both comorbidity management and CSU treatment.

## 9. Clinical Implications and Future Directions

The heterogeneity of CSU underscores the need for individualized assessment of comorbidities and potential disease modifiers (Table 2). Comorbidities may provide clinically relevant information about underlying disease biology, factors modifying disease activity, and the broader burden of CSU. Their clinical significance, however, depends on whether they reflect shared susceptibility, act as disease modifiers, or emerge as downstream consequences of persistent disease. Treatment-related morbidity should also be distinguished from comorbidities associated with the underlying disease. These distinctions may help guide individualized clinical assessment and management (Table 2).

In routine practice, assessment of CSU-associated comorbidities should be guided by clinical history, symptoms, physical examination, and established risk factors rather than by universal screening. Follow-up should focus on changes in disease control, newly emerging symptoms, and treatment-related adverse effects, with reassessment of associated conditions when clinically indicated. In the absence of specific clinical indications, current evidence does not support routine screening for malignancy, chronic infections, or other systemic disorders solely on the basis of a CSU diagnosis [1].

Future studies should move beyond cross-sectional associations to better define the temporal and mechanistic relationships between CSU and its comorbidities. Longitudinal studies are needed to determine which conditions precede CSU, which emerge as consequences of persistent disease, and which are involved in bidirectional inflammatory amplification. Clarifying these relationships may facilitate more individualized treatment approaches. Finally, prospective studies should determine whether earlier and more effective CSU control can modify not only disease activity but also the development or course of associated comorbidities and long-term systemic outcomes.

## 10. Conclusions

Comorbidities are integral to understanding and managing CSU. They may reflect underlying disease susceptibility, trigger or modify disease activity, or emerge as downstream consequences of persistent disease and its treatment. Their age-dependent, temporal, and multidirectional relationships further suggest that the distinctions among disease drivers, modifiers, and systemic consequences are not always clear.

A comprehensive assessment of comorbidities may therefore provide insight into CSU heterogeneity while identifying broader health risks relevant to patient management. Integrating comorbidity assessment into CSU care may help refine disease phenotyping, guide individualized management, and address the systemic burden extending beyond wheals and angioedema.

## Figures and Tables

**Table 1:** Epidemiologic burden and associations of major comorbidities in chronic spontaneous urticaria.

Domain	Comorbidity	Prevalence in CU/CSU	Association/Key Estimate	Study Design	Ref.
Autoimmune	Thyroid autoimmunity	—	Anti-TPO positivity: approximately fivefold higher odds than in controls	Meta-analysis of case–control studies	[4]
	Autoimmune diseases overall	—	Increased occurrence of RA, Sjögren syndrome, celiac disease, type 1 diabetes, and SLE; several diagnosed predominantly within 10 years after CU diagnosis	Population-based database study	[5]
Atopic	Allergic rhinitis	~15% to >50%	Most frequently reported atopic comorbidity	Observational studies; systematic review/meta-analysis	[6,7,8,9,10,11,12,13,14,15]
	Asthma	~10–40%	More prominent in younger patients		
	Atopic dermatitis	~5% to >20%	More prominent in children and younger patients		
	Anaphylaxis	—	aHR 2.53	Nationwide population-based longitudinal studies	[16,17,18]
Psychiatric	Any psychiatric disorder	~1 in 3 patients	—	Systematic review/meta-analysis	[19]
	Depression	48.1% ^a^	OR 1.50 (95% CI, 1.30–1.70) vs. matched controls	Multicenter observational study using HADS (prevalence); nationwide registry study (OR)	[16,20]
	Anxiety	38.0% ^a^	OR 1.10 (95% CI, 1.04–1.16) vs. non-atopic eczema	Multicenter observational study using HADS (prevalence); claims-based cross-sectional study (OR)	[20,21]
	Sleep disorder	—	OR 1.18 (95% CI, 1.11–1.26) vs. non-atopic eczema	Claims-based cross-sectional study	[21]
	Suicidal ideation	—	OR 1.33 (95% CI, 1.15–1.53) vs. non-atopic eczema		
Metabolic	Metabolic syndrome	25.2% pooled; 29.8% in a Korean cohort (17.8% in controls)	~1.6-fold higher odds than in controls; associated with uncontrolled urticaria	Systematic review/meta-analysis; hospital-based cross-sectional study	[22,23]
Cardiovascular	Major adverse cardiovascular events	—	aHR 1.25	Propensity score-matched cohort study	[24]
	Venous thromboembolism	—	aHR 1.63		
	Conduction disorders	—	aHR 1.82		
	Atherosclerotic cardiovascular disease	—	Independently associated after adjustment for cardiovascular risk factors	Nationwide claims-based cohort study	[25]
Infection/vaccination	*Helicobacter pylori* infection	—	Lower spontaneous remission in *H. pylori*-positive patients; eradication therapy associated with higher remission, independent of eradication success	Meta-analysis	[26,27]
	Chronic hepatitis B/C	HBV ≤ 5%; HCV ≤ 2% in most studies	Generally comparable to rates in the general population	Systematic review	[28]
	COVID-19	—	Worsening in 37% of patients with pre-existing CU; incident CU: aHR 1.80 (95% CI, 1.64–1.98) and HR 1.13	International cohort; population-based cohort studies	[29,30,31]
	Vaccination	—	ROR 26.52 (95% CI, 24.33–28.90) for COVID-19 mRNA vaccines	Global pharmacovigilance (VigiBase) disproportionality analysis	[32]
Malignancy and mortality	Malignancy	—	Inconsistent across populations	Population-based cohort study	[33,34,35,36]
	All-cause mortality	—	Higher at 3 months, 1 year, and 5 years than in matched controls	Propensity score-matched real-world cohort study	[37]

Estimates are drawn from studies discussed in this review and were not identified through a systematic search; values are not pooled across studies. The table is limited to comorbidities for which quantitative estimates were reported. —, not specifically addressed in the present review. ^a^ Determined by the Hospital Anxiety and Depression Scale (HADS), a screening instrument, and therefore not equivalent to a clinical diagnosis. Abbreviations: aHR, adjusted hazard ratio; CI, confidence interval; CSU, chronic spontaneous urticaria; CU, chronic urticaria; HADS, Hospital Anxiety and Depression Scale; HBV, hepatitis B virus; HCV, hepatitis C virus; HR, hazard ratio; OR, odds ratio; RA, rheumatoid arthritis; ROR, reporting odds ratio; SLE, systemic lupus erythematosus; TPO, thyroid peroxidase.

**Table 2:** Practical approach to comorbidity assessment and management in chronic spontaneous urticaria.

Domain	Suggested Assessment	Who/When to Assess	Clinical Rationale	Practical Implication
Autoimmune	Basic CSU workup: differential blood count and CRP and/or ESR; in specialist care, total IgE and IgG anti-TPO. Thyroid function and targeted autoimmune testing when clinically indicated. Selected use of ASST or basophil-based testing may support type IIb endotyping.	All patients at initial evaluation; additional testing guided by history, examination, and longstanding or uncontrolled disease.	Thyroid autoimmunity is the best-established autoimmune association and may indicate an autoimmune-prone milieu. Type IIb CSU is associated with characteristic laboratory and clinical features.	Avoid indiscriminate autoantibody panels. Treat coexisting autoimmune disease; advanced type IIb assays remain limited to specialist or research settings.
Atopic/allergic	Structured history of allergic rhinitis, asthma, atopic dermatitis, food allergy, previous systemic reactions, and relevant cofactors (including NSAIDs); allergen testing only when clinically indicated.	History in all patients; focused testing when symptoms or exposure history suggest an allergic disorder.	Atopic diseases frequently coexist with CSU, with age- and population-dependent patterns. Associations with food allergy and anaphylaxis may reflect shared allergic or mast cell susceptibility.	Atopy or allergen sensitization alone does not define type I autoimmune (autoallergic) CSU; avoid broad allergy panels without a relevant history.
Neuroimmune/psychiatric	Brief inquiry regarding anxiety, depressive symptoms, sleep disturbance, and disease-related distress; HADS, PSQI, or equivalent instruments when appropriate. Assess QoL and CSU activity/control (e.g., UAS7 and UCT).	Brief clinical inquiry in all patients; formal assessment especially in those with persistent or uncontrolled disease, sleep disturbance, impaired QoL, or clinical concern.	Psychological burden is common, and itch, sleep loss, and stress may form bidirectional neuroimmune loops. Psychiatric or sleep symptoms may persist despite improvement in urticaria.	Assess and manage psychiatric and sleep comorbidities independently of CSU control; refer when appropriate.
Metabolic/cardiovascular	BMI and blood pressure; glucose and lipid assessment according to age, risk factors, and general preventive-care recommendations.	Adults, particularly those with obesity or established cardiometabolic risk factors.	Metabolic syndrome and cardiovascular outcomes are associated with CU/CSU in observational studies; low-grade inflammation, endothelial dysfunction, and coagulation abnormalities provide plausible links, but causality remains uncertain.	Follow general cardiometabolic prevention standards.
Infections and vaccination	Focused history of recent or recurrent infections, gastrointestinal symptoms, and flares temporally related to infection (including COVID-19) or vaccination. Infection testing (e.g., *H. pylori*) guided by history and symptoms; HBV/HCV serology only in patients with risk factors or abnormal liver function.	History in all patients; testing only when history, symptoms, laboratory abnormalities, or a flare temporally related to infection or vaccination suggest relevance.	Infections and vaccination may act as triggers or modifiers in susceptible patients rather than as common underlying causes. *H. pylori* eradication has been associated with remission, but this association appears to be independent of successful bacterial eradication; chronic viral hepatitis is not more prevalent than in the general population. SARS-CoV-2 infection and vaccination may precipitate new-onset CU or flares by amplifying pre-existing autoimmune or autoallergic pathways.	Avoid routine infection screening; treat identified infections according to standard indications. A post-vaccination flare does not by itself indicate vaccine allergy, and CSU alone is not a contraindication to vaccination.
Malignancy/mortality	No CSU-specific routine cancer screening; age-appropriate screening and directed evaluation for red flags such as unexplained lymphadenopathy, weight loss, persistent fever, or other concerning systemic findings.	Directed evaluation for red-flag presentations; otherwise, routine population-based screening.	Reported malignancy associations are inconsistent; recent mortality signals may reflect underlying comorbidity burden rather than a direct effect of CU.	CSU alone does not warrant additional malignancy screening beyond general population recommendations.
Treatment-related	Review cumulative systemic corticosteroid exposure; apply treatment-specific safety monitoring (e.g., blood pressure and renal function with cyclosporine); assess metabolic, bone, and infection risk when corticosteroid exposure is recurrent or prolonged.	According to treatment exposure, duration, and cumulative burden.	Treatment-related toxicity can add metabolic, bone, infectious, renal, and functional morbidity to the underlying disease burden.	Minimize cumulative systemic corticosteroid exposure and apply guideline-directed, treatment-specific safety monitoring.

Abbreviations: ASST, autologous serum skin test; BMI, body mass index; CRP, C-reactive protein; CSU, chronic spontaneous urticaria; CU, chronic urticaria; ESR, erythrocyte sedimentation rate; HADS, Hospital Anxiety and Depression Scale; HBV, hepatitis B virus; HCV, hepatitis C virus; Ig, immunoglobulin; NSAID, nonsteroidal anti-inflammatory drug; PSQI, Pittsburgh Sleep Quality Index; QoL, quality of life; TPO, thyroid peroxidase; UAS7, Urticaria Activity Score over 7 days; UCT, Urticaria Control Test.

## Data Availability

No new data were created or analyzed in this study. Data sharing is not applicable to this article.

## References

[1] Zuberbier T., Ansari Z.A., Abdul Latiff A.H., Abuzakouk M.M., Agcaoili-De Jesus M.S., Agondi R.C., Al-Ahmad M., Alangari A.A., Alhameli H., Alonso Bello C.D. (2026). The International Guideline for the Definition, Classification, Diagnosis and Management of Urticaria. Allergy.

[2] Yang S., Chen L., Zhang H., Song Y., Wang W., Hu Z., Wang S., Huang L., Wang Y., Wu S. (2025). Beyond the itch: The complex interplay of immune, neurological, and psychological factors in chronic urticaria. J. Neuroinflamm..

[3] Kolkhir P., Borzova E., Grattan C., Asero R., Pogorelov D., Maurer M. (2017). Autoimmune comorbidity in chronic spontaneous urticaria: A systematic review. Autoimmun. Rev..

[4] Tienforti D., Di Giulio F., Spagnolo L., Castellini C., Totaro M., Muselli M., Francavilla S., Baroni M.G., Barbonetti A. (2022). Chronic urticaria and thyroid autoimmunity: A meta-analysis of case-control studies. J. Endocrinol. Investig..

[5] Confino-Cohen R., Chodick G., Shalev V., Leshno M., Kimhi O., Goldberg A. (2012). Chronic urticaria and autoimmunity: Associations found in a large population study. J. Allergy Clin. Immunol..

[6] Papapostolou N., Xepapadaki P., Katoulis A., Makris M. (2022). Comorbidities of Chronic Urticaria: A glimpse into a complex relationship. Front. Allergy.

[7] Weller K., Maurer M., Bauer A., Wedi B., Wagner N., Schliemann S., Kramps T., Baeumer D., Multmeier J., Hillmann E. (2022). Epidemiology, comorbidities, and healthcare utilization of patients with chronic urticaria in Germany. J. Eur. Acad. Dermatol. Venereol..

[8] Lachover-Roth I., Rabie A., Cohen-Engler A., Rosman Y., Meir-Shafrir K., Confino-Cohen R. (2021). Chronic urticaria in children—New insights from a large cohort. Pediatr. Allergy Immunol..

[9] Ha E.K., Kim J.H., Shin J., Lee W.S., Han B., Lee E., Rhie S., Kim D.H., Han M.Y. (2025). Lifetime Age-Stratified Associations of Comorbidities in Chronic Urticaria: A Claims-Based Study of 35,907 Cases. Clin. Exp. Allergy.

[10] Fukunaga A., Kishi Y., Sunaga Y., Arima K. (2025). Prevalence, comorbidities, and current management of chronic spontaneous urticaria in Japan: Retrospective claims database study. J. Dermatol..

[11] Xiao M., Zhang X., Guo A., Zhang K., Zhao Q., Fan L., Xue L., Zhang J., Hou S., Wang H. (2024). A Retrospective Cohort Study on the Comorbidity in 19,264 Chinese Patients of Different Ages with Urticaria. Acta Derm. Venereol..

[12] Cortes J.A., Adjei-Frimpong N.A., Fakhouri S.K., Delacqua F., Oldenburg R. (2026). Atopic Comorbidities Associated With Chronic Spontaneous Urticaria: A Case-Control Analysis of the All of Us Research Program. JEADV Clin. Pract..

[13] Toubi E., Mubariki R., Vadasz Z. (2025). Co-existence of chronic spontaneous urticaria with atopic dermatitis: Clinical and immunological perspectives. Front. Allergy.

[14] Zhang D.G., Zahid J.A., Ali Z., Thomsen S.F. (2023). Risk of Atopic Disorders in Patients with Chronic Urticaria: A Systematic Review and Meta-Analysis. Dermatology.

[15] Thomsen S., Pritzier E., Anderson C., Vaugelade-Baust N., Dodge R., Dahlborn A.K., Vestergaard C. (2017). Chronic urticaria in the real-life clinical practice setting in Sweden, Norway and Denmark: Baseline results from the non-interventional multicentre AWARE study. J. Eur. Acad. Dermatol. Venereol..

[16] Ghazanfar M.N., Kibsgaard L., Thomsen S.F., Vestergaard C. (2020). Risk of comorbidities in patients diagnosed with chronic urticaria: A nationwide registry-study. World Allergy Organ. J..

[17] Kim J.H., Lee E., Shin J., Ha E., Yoo H., Lee S., Han M.Y. (2026). Age-stratified comorbidity transitions and interconnected mapping of nine allergic diseases. Allergol. Int..

[18] Yong S.B., Chen H.H., Huang J.Y., Wei J.C. (2018). Patients with urticaria are at a higher risk of anaphylaxis: A nationwide population-based retrospective cohort study in Taiwan. J. Dermatol..

[19] Konstantinou G.N., Konstantinou G.N. (2019). Psychiatric comorbidity in chronic urticaria patients: A systematic review and meta-analysis. Clin. Transl. Allergy.

[20] Choi G.S., Nam Y.H., Park C.S., Kim M.Y., Jo E.J., Park H.K., Kim H.K. (2020). Anxiety, depression, and stress in Korean patients with chronic urticaria. Korean J. Intern. Med..

[21] Ahn H.J., Shin M.K., Seo J.K., Jeong S.J., Cho A.R., Choi S.H., Lew B.L. (2019). Cross-sectional study of psychiatric comorbidities in patients with atopic dermatitis and nonatopic eczema, urticaria, and psoriasis. Neuropsychiatr. Dis. Treat..

[22] Ye Y.M., Jin H.J., Hwang E.K., Nam Y.H., Kim J.H., Shin Y.S., Park H.S. (2013). Co-existence of chronic urticaria and metabolic syndrome: Clinical implications. Acta Derm. Venereol..

[23] Rayner D.G., Gou D., Sun W. (2025). Prevalence of Metabolic Syndrome in Chronic Urticaria: A Systematic Review and Meta-Analysis. Clin. Exp. Allergy.

[24] Curman P., Thaçi D., Ludwig R.J. (2026). Increased Cardiovascular Risk in Chronic Spontaneous Urticaria: A Large Real-World Cohort Study. Allergy.

[25] Cortes J.A., Nong Y., Smart K., Aflatooni S., Palma G., Johnsen N., Ure A., Armstrong A.W. (2026). Chronic Urticaria Is Independently Associated with Atherosclerotic Cardiovascular Disease: A National Cohort Study of the MarketScan Databases. Am. J. Clin. Dermatol..

[26] Kim H.J., Kim Y.J., Lee H.J., Hong J.Y., Park A.Y., Chung E.H., Lee S.Y., Lee J.S., Park Y.L., Lee S.H. (2019). Systematic review and meta-analysis: Effect of Helicobacter pylori eradication on chronic spontaneous urticaria. Helicobacter.

[27] Federman D.G., Kirsner R.S., Moriarty J.P., Concato J. (2003). The effect of antibiotic therapy for patients infected with *Helicobacter pylori* who have chronic urticaria. J. Am. Acad. Dermatol..

[28] Kolkhir P., Pereverzina N., Olisova O., Maurer M. (2018). Comorbidity of viral hepatitis and chronic spontaneous urticaria: A systematic review. Allergy.

[29] Kocaturk E., Salman A., Cherrez-Ojeda I., Criado P.R., Peter J., Comert-Ozer E., Abuzakouk M., Agondi R.C., Al-Ahmad M., Altrichter S. (2021). The global impact of the COVID-19 pandemic on the management and course of chronic urticaria. Allergy.

[30] Lee S., Park J., Kang J., Smith L., Rahmati M., Lee H., Yon D.K. (2024). Risks of chronic urticaria after SARS-CoV-2 infection: Binational population-based cohort studies from South Korea and Japan. J. Allergy Clin. Immunol. Pract..

[31] Kim M.H. (2025). Epidemiological insights into chronic urticaria, vitiligo, alopecia areata, and herpes zoster following COVID-19 infection: A nationwide population-based study. J. Dermatol..

[32] Kang J., Park J., Jo H., Lee H., Lee K., Kim S., Kang J., Lee J.H., Papadopoulos N.G., Smith L. (2024). Global Burden of Vaccine-Associated Chronic Urticaria, 2010-2023: From the Global Pharmacovigilance Database. Allergy Asthma Immunol. Res..

[33] Kim B.R., Yang S., Choi J.W., Choi C.W., Youn S.W. (2018). Epidemiology and comorbidities of patients with chronic urticaria in Korea: A nationwide population-based study. J. Dermatol..

[34] Chu C.Y., Cho Y.T., Jiang J.H., Lin E.I., Tang C.H. (2017). Epidemiology and comorbidities of patients with chronic urticaria in Taiwan: A nationwide population-based study. J. Dermatol. Sci..

[35] Sorensen S.B.T., Farkas D.K., Vestergaard C., Schmidt S.A.J., Lindahl L.M., Mansfield K.E., Langan S.M., Sorensen H.T. (2024). Urticaria and the risk of cancer: A Danish population-based cohort study. Br. J. Dermatol..

[36] Chen Y.J., Wu C.Y., Shen J.L., Chen T.T., Chang Y.T. (2012). Cancer risk in patients with chronic urticaria: A population-based cohort study. Arch. Dermatol..

[37] Kolkhir P., Bieber K., Hawro T., Kridin K., Ludwig M.A., Olbrich H., Metz M., Vorobyev A., Ludwig R.J., Maurer M. (2025). Mortality in adult patients with chronic spontaneous urticaria: A real-world cohort study. J. Allergy Clin. Immunol..

[38] Sun N., Ogulur I., Mitamura Y., Yazici D., Pat Y., Bu X., Li M., Zhu X., Babayev H., Ardicli S. (2024). The epithelial barrier theory and its associated diseases. Allergy.

[39] Ye Y.M. (2026). Targeting immune dysregulation in chronic spontaneous urticaria: Beyond antihistamines. Korean J. Intern. Med..

[40] Zhuang L., Zhuang C., Jiang H., Su S. (2026). Thyroid autoantibodies predict long-term treatment response in euthyroid chronic spontaneous urticaria: A retrospective cohort study with propensity score matching. Front. Med..

[41] Kolkhir P., Altrichter S., Asero R., Daschner A., Ferrer M., Giménez-Arnau A., Hawro T., Jakob T., Kinaciyan T., Kromminga A. (2021). Autoimmune Diseases Are Linked to Type IIb Autoimmune Chronic Spontaneous Urticaria. Allergy Asthma Immunol. Res..

[42] Kolkhir P., Munoz M., Asero R., Ferrer M., Kocaturk E., Metz M., Xiang Y.K., Maurer M. (2022). Autoimmune chronic spontaneous urticaria. J. Allergy Clin. Immunol..

[43] Schoepke N., Asero R., Ellrich A., Ferrer M., Gimenez-Arnau A., Clive E.H.G., Jakob T., Konstantinou G.N., Raap U., Skov P.S. (2019). Biomarkers and clinical characteristics of autoimmune chronic spontaneous urticaria: Results of the PURIST Study. Allergy.

[44] Kolkhir P., Metz M., Altrichter S., Maurer M. (2017). Comorbidity of chronic spontaneous urticaria and autoimmune thyroid diseases: A systematic review. Allergy.

[45] Choi B.Y., Yang E.M., Jung H.W., Shin M.K., Jo J., Cha H.Y., Park H.S., Kang H.C., Ye Y.M. (2023). Anti-heat shock protein 10 IgG in chronic spontaneous urticaria: Relation with miRNA-101-5p and platelet-activating factor. Allergy.

[46] Yanase Y., Takahagi S., Ozawa K., Hide M. (2021). The Role of Coagulation and Complement Factors for Mast Cell Activation in the Pathogenesis of Chronic Spontaneous Urticaria. Cells.

[47] Asero R., Tedeschi A., Coppola R., Griffini S., Paparella P., Riboldi P., Marzano A.V., Fanoni D., Cugno M. (2007). Activation of the tissue factor pathway of blood coagulation in patients with chronic urticaria. J. Allergy Clin. Immunol..

[48] Alizadeh Aghdam M., van den Elzen M., van Os-Medendorp H., van Dijk M.R., Knol E.F., Knulst A.C., Röckmann H., Otten H.G. (2021). Systemic and local evidence for complement involvement in chronic spontaneous urticaria. Clin. Transl. Allergy.

[49] Ferrer M., Nakazawa K., Kaplan A.P. (1999). Complement dependence of histamine release in chronic urticaria. J. Allergy Clin. Immunol..

[50] Wong D., Waserman S., Sussman G.L. (2025). Endotypes of chronic spontaneous urticaria and angioedema. J. Allergy Clin. Immunol..

[51] Schmetzer O., Lakin E., Topal F.A., Preusse P., Freier D., Church M.K., Maurer M. (2018). IL-24 is a common and specific autoantigen of IgE in patients with chronic spontaneous urticaria. J. Allergy Clin. Immunol..

[52] Jang J.H., Xiang Y.K., Yang E.M., Kim J.H., Choi B., Park H.S., Maurer M., Ye Y.M. (2024). Distinct Clinical Profiles of IgE and IgG Autoantibodies to Thyroid Peroxidase in Chronic Spontaneous Urticaria. Allergy Asthma Immunol. Res..

[53] Cugno M., Asero R., Ferrucci S., Lorini M., Carbonelli V., Tedeschi A., Marzano A.V. (2018). Elevated IgE to tissue factor and thyroglobulin are abated by omalizumab in chronic spontaneous urticaria. Allergy.

[54] Xiang Y.K., Kolkhir P., Scheffel J., Sauer M., Vera C., Frischbutter S., Krause K., Siebenhaar F., Metz M., Maurer M. (2023). Most Patients With Autoimmune Chronic Spontaneous Urticaria Also Have Autoallergic Urticaria, but Not ViceVersa. J. Allergy Clin. Immunol. Pract..

[55] Sánchez J., Sánchez A., Cardona R. (2019). Causal Relationship Between Anti-TPO IgE and Chronic Urticaria by In Vitro and In Vivo Tests. Allergy Asthma Immunol. Res..

[56] Kitsioulis N.A., Papadopoulos N.G., Kostoudi S., Manousakis E., Douladiris N., Xepapadaki P. (2018). Assessment of atopic dermatitis as a risk factor for chronic spontaneous urticaria in a pediatric population. Allergy Asthma Proc..

[57] Geha R.S., Jabara H.H., Brodeur S.R. (2003). The regulation of immunoglobulin E class-switch recombination. Nat. Rev. Immunol..

[58] Toru H., Ra C., Nonoyama S., Suzuki K., Yata J., Nakahata T. (1996). Induction of the high-affinity IgE receptor (Fc epsilon RI) on human mast cells by IL-4. Int. Immunol..

[59] Chen Q., Wang W., Yang X., Li S., Deng S., Wang H., Liu W., Ni B., Song Z. (2024). Characteristics and Clinical Significance of Atopy in Chronic Spontaneous Urticaria: A Cross-Sectional Observational Study. Int. Arch. Allergy Immunol..

[60] Konstantinou G.N., Konstantinou G.N. (2020). Psychological Stress and Chronic Urticaria: A Neuro-immuno-cutaneous Crosstalk. A Systematic Review of the Existing Evidence. Clin. Ther..

[61] Wang Z., Franke K., Bal G., Li Z., Zuberbier T., Babina M. (2022). MRGPRX2-Mediated Degranulation of Human Skin Mast Cells Requires the Operation of G_αi_, G_αq_, Ca++ Channels, ERK1/2 and PI3K—Interconnection between Early and Late Signaling. Cells.

[62] Metz M., Krull C., Hawro T., Saluja R., Groffik A., Stanger C., Staubach P., Maurer M. (2014). Substance P is upregulated in the serum of patients with chronic spontaneous urticaria. J. Investig. Dermatol..

[63] Memet B., Vurgun E., Barlas F., Metz M., Maurer M., Kocatürk E. (2021). In chronic spontaneous urticaria, comorbid depression linked to higher disease activity, and substance P levels. Front. Psychiatry.

[64] Boyvadoglu C., Ulusal H., Taysi S., Ozaydin-Yavuz G., Yavuz I.H., Korkmaz P., Inaloz H.S. (2023). Effects of Omalizumab on Serum Levels of Substance P, Calcitonin Gene-Related Peptide, Neuropeptide Y, and Interleukin-31 in Patients with Chronic Spontaneous Urticaria. Mediat. Inflamm..

[65] Chaowattanapanit S., Choonhakarn C., Salao K., Winaikosol K., Julanon N., Wongjirattikarn R., Foocharoen C., Sompornrattanaphan M. (2020). Increased serum IL-31 levels in chronic spontaneous urticaria and psoriasis with pruritic symptoms. Heliyon.

[66] Rössing K., Novak N., Mommert S., Pfab F., Gehring M., Wedi B., Kapp A., Raap U. (2011). Brain-derived neurotrophic factor is increased in serum and skin levels of patients with chronic spontaneous urticaria. Clin. Exp. Allergy.

[67] Zhao Y., Yang H., Yu S., Li Y., Zhang L. (2025). Pruritus and anxiety symptoms in chronic spontaneous urticaria are associated with altered functional connectivity of the brain. J. Neuroradiol..

[68] Patella V., Zunno R., Florio G., Palmieri M., Palmieri S., Brancaccio R. (2021). Omalizumab improves perceived stress, anxiety, and depression in chronic spontaneous urticaria. J. Allergy Clin. Immunol. Pract..

[69] Esen M., Demirbas A., Diremsizoglu E. (2025). Quality of life, sleep, and psychological well-being in chronic spontaneous urticaria patients receiving omalizumab: A case-control study. Arch. Dermatol. Res..

[70] Eskeland S., Halvorsen J.A., Tanum L. (2017). Antidepressants have Anti-inflammatory Effects that may be Relevant to Dermatology: A Systematic Review. Acta Derm. Venereol..

[71] Maged Amin M., Rushdy M. (2018). Hyperlipidemia in association with pro-inflammatory cytokines among chronic spontaneous urticaria: Case-control study. Eur. Ann. Allergy Clin. Immunol..

[72] Kasperska-Zajac A., Sztylc J., Machura E., Jop G. (2011). Plasma IL-6 concentration correlates with clinical disease activity and serum C-reactive protein concentration in chronic urticaria patients. Clin. Exp. Allergy.

[73] Vena G.A., Cassano N. (2017). The link between chronic spontaneous urticaria and metabolic syndrome. Eur. Ann. Allergy Clin. Immunol..

[74] Mostmans Y., De Smedt K., Richert B., Elieh Ali Komi D., Maurer M., Michel O. (2021). Markers for the involvement of endothelial cells and the coagulation system in chronic urticaria: A systematic review. Allergy.

[75] Chung S.D., Wang K.H., Tsai M.C., Lin H.C., Chen C.H. (2016). Hyperlipidemia Is Associated with Chronic Urticaria: A Population-Based Study. PLoS ONE.

[76] Egeberg A., Kofoed K., Gislason G.H., Vestergaard C., Thyssen J.P. (2017). Cardiovascular Risk is not Increased in Patients with Chronic Urticaria: A Retrospective Population-based Cohort Study. Acta Derm. Venereol..

[77] Wang C.W., Chen C.B., Lu C.W., Chen W.T., Hui R.C., Chiu T.M., Chi M.H., Lin J.C., Huang Y.H., Chang Y.C. (2023). Characteristics of immune response profile in patients with immediate allergic and autoimmune urticarial reactions induced by SARS-CoV-2 vaccines. J. Autoimmun..

[78] Magen E., Yakov A., Green I., Israel A., Vinker S., Merzon E. (2022). Chronic spontaneous urticaria after BNT162b2 mRNA (Pfizer-BioNTech) vaccination against SARS-CoV-2. Allergy Asthma Proc..

[79] Ye Y.M. (2024). Can Vaccinations Trigger or Exacerbate Chronic Urticaria?. Allergy Asthma Immunol. Res..

[80] Grivennikov S.I., Greten F.R., Karin M. (2010). Immunity, inflammation, and cancer. Cell.

[81] Lekli E., Hoxha M., Bova M., Gjonbrataj J., Mati K., Vyshka G., Qirko E. (2026). Chronic Urticaria and Malignancy: A Review Uncovering the Common Links. Biomedicines.

[82] Larenas-Linnemann D., Saini S.S., Azamar-Jacome A.A., Maurer M. (2018). Chronic urticaria can be caused by cancer and resolves with its cure. Allergy.

[83] Sanchez-Borges M., Ansotegui I.J., Baiardini I., Bernstein J., Canonica G.W., Ebisawa M., Gomez M., Gonzalez-Diaz S.N., Martin B., Morais-Almeida M. (2021). The challenges of chronic urticaria part 1: Epidemiology, immunopathogenesis, comorbidities, quality of life, and management. World Allergy Organ. J..

[84] Ledford D., Broder M.S., Antonova E., Omachi T.A., Chang E., Luskin A. (2016). Corticosteroid-related toxicity in patients with chronic idiopathic urticaria–chronic spontaneous urticaria. Allergy Asthma Proc..

[85] Ye Y.M., Koh Y.I., Choi J.H., Kim M.A., Park J.W., Kim T.B., Nam Y.H., Chang Y.S., Park H.S. (2022). The burden of symptomatic patients with chronic spontaneous urticaria: A real-world study in Korea. Korean J. Intern. Med..

[86] Maurer M., Abuzakouk M., Berard F., Canonica W., Oude Elberink H., Gimenez-Arnau A., Grattan C., Hollis K., Knulst A., Lacour J.P. (2017). The burden of chronic spontaneous urticaria is substantial: Real-world evidence from ASSURE-CSU. Allergy.

[87] Berberi G., Maazi M., Prosty C., McMullen E.P., Fein M., Lima H., Martinez-Jaramillo E., Ben-Shoshan M., Pourpanah F., Netchiporouk E. (2025). Health-Related Quality of Life in Chronic Urticaria: A Systematic Review and Meta-Analysis. J. Allergy Clin. Immunol. Pract..

[88] Augustin M., Beier D., Branner J., Hackl D., Hampel R., Kramps T., Kurzen H., Lintener H., Melzer N., Muller M. (2024). The socio-economic burden of H1-antihistamine-refractory chronic spontaneous urticaria in Germany. J. Eur. Acad. Dermatol. Venereol..

